# Effects of a large-scale, natural sediment deposition event on plant cover in a Massachusetts salt marsh

**DOI:** 10.1371/journal.pone.0245564

**Published:** 2021-01-22

**Authors:** G. E. Moore, D. M. Burdick, M. R. Routhier, A. B. Novak, A. R. Payne

**Affiliations:** 1 Jackson Estuarine Laboratory, School of Marine Science and Ocean Engineering, University of New Hampshire, Durham, New Hampshire, United States of America; 2 Geospatial Science Center Earth Systems Research Center, Institute for the Study of Earth, Oceans, and Space, University of New Hampshire, Durham, New Hampshire, United States of America; 3 Earth and Environment Department, Boston University, Boston, Massachusetts, United States of America; Shandong University, CHINA

## Abstract

In mid-winter 2018, an unprecedented sediment deposition event occurred throughout portions of the Great Marsh in Massachusetts. Evaluation of this event in distinct marsh areas spanning three towns (Essex, Ipswich, and Newbury) revealed deposition covering 29.2 hectares with an average thickness of 30.1±2.1 mm measured shortly after deposition. While sediment deposition helps marshes survive sea level rise by building elevation, effects of such a large-scale deposition on New England marshes are unknown. This natural event provided an opportunity to study effects of large-scale sediment addition on plant cover and soil chemistry, with implications for marsh resilience. Sediment thickness did not differ significantly between winter and summer, indicating sediment is not eroding or compacting. The deposited sediment at each site had similar characteristics to that of the adjacent mudflat (e.g., texture, bivalve shells), suggesting that deposited materials resulted from ice rafting from adjacent flats, a natural phenomenon noted by other authors. Vegetative cover was significantly lower in plots with rafted sediment (75.6±2.3%) than sediment-free controls (93.1±1.6%) after one growing season. When sorted by sediment thickness categories, the low thickness level (1–19 mm) had significantly greater percent cover than medium (20–39 mm) and high (40–90 mm) categories. Given that sediment accretion in the Great Marsh was found to average 2.7 mm per year, the sediment thickness documented herein represents ~11 years of sediment accretion with only a 25% reduction in plant cover, suggesting this natural sediment event will likely increase long-term marsh resilience to sea level rise.

## Introduction

Salt marshes, widely recognized for their valuable suite of ecosystem services (flood protection, habitat provision, carbon storage, etc. [1, 2]), are both dependent upon tidal flooding but also vulnerable to increased rates of sea level rise [3, 4]. Marshes undergo constant change as they respond to both hydrological and biological processes; tidal flooding supplies marshes with sediment, while halophytic plants help to trap sediment [5] and build peat through the input of organic matter [6, 7]. Salt marshes can be thought of as poised systems reflecting a balance of sediment accretion and peat development on one side versus erosion, compaction and oxidation of sediments on the other [5, 8]. Human actions, ranging in scale from local (e.g., ditching, impoundments) to global (e.g., sea level rise), have disrupted this balance, resulting in marsh loss and conversion of high marsh to low marsh [9, 10]. As average water levels rise, the potential for sediments suspended by flooding tides to deposit on the marsh increases, and the potential for peat to dry out and oxidize decreases. Over time this deposited sediment, combined with accumulation of organic matter leads to elevation gains on the marsh surface, but marsh elevation gain is lagging behind sea level rise in many marshes [3, 11, 12].

Given the growing concern of marsh loss, conversion of high marsh to low marsh with elevated sea levels [9, 10], and impediments to sediment supply processes that help increase marsh elevation [13], natural events that supply sediment to the marsh surface are particularly valuable. Although storm waves can erode marsh edges [14], deposition events resulting from storms can build the elevation of submerging marshes and stimulate plant growth [15, 16]. Sediment deposition from Hurricanes has been well-documented, particularly in southern marshes [15, 16], but also in New England [17]. Another important mechanism for sediment deposition in northern marshes is ice rafting [18–20]. Deposition through ice rafting occurs when ice forms and freezes to exposed mudflats at low tide, and the rising tide transports the ice sheets to the adjacent marsh, along with mudflat sediment attached to the bottom of the ice [18]. When the ice melts, any sediment contained within or beneath the ice is left on the marsh surface. This process can be repeated throughout the winter, resulting in small patches of sediment accumulation on the marsh surface (up to 10.5 m^2^), sometimes as multiple layers [18].

Because sediment deposition has been shown to improve plant growth and may increase resilience to sea-level rise, the process has been replicated artificially through thin-layer placement (TLP), which typically involves jet-spraying a slurry of dredged material onto the marsh [21]. The use of TLP as a restoration tool has occurred in some mid to south-Atlantic and Gulf states where marsh loss and degradation have been rapid and extreme, particularly in the low-lying Mississippi Delta of Louisiana. Rhode Island is the only state in New England that has applied TLP to marshes on a larger scale, though results are not yet available. In Massachusetts, current wetland regulations prevent the use of any material to raise marsh elevation, making it impossible to study effects of large-scale TLP unless the sediment deposition occurs naturally.

**Fig 1:**
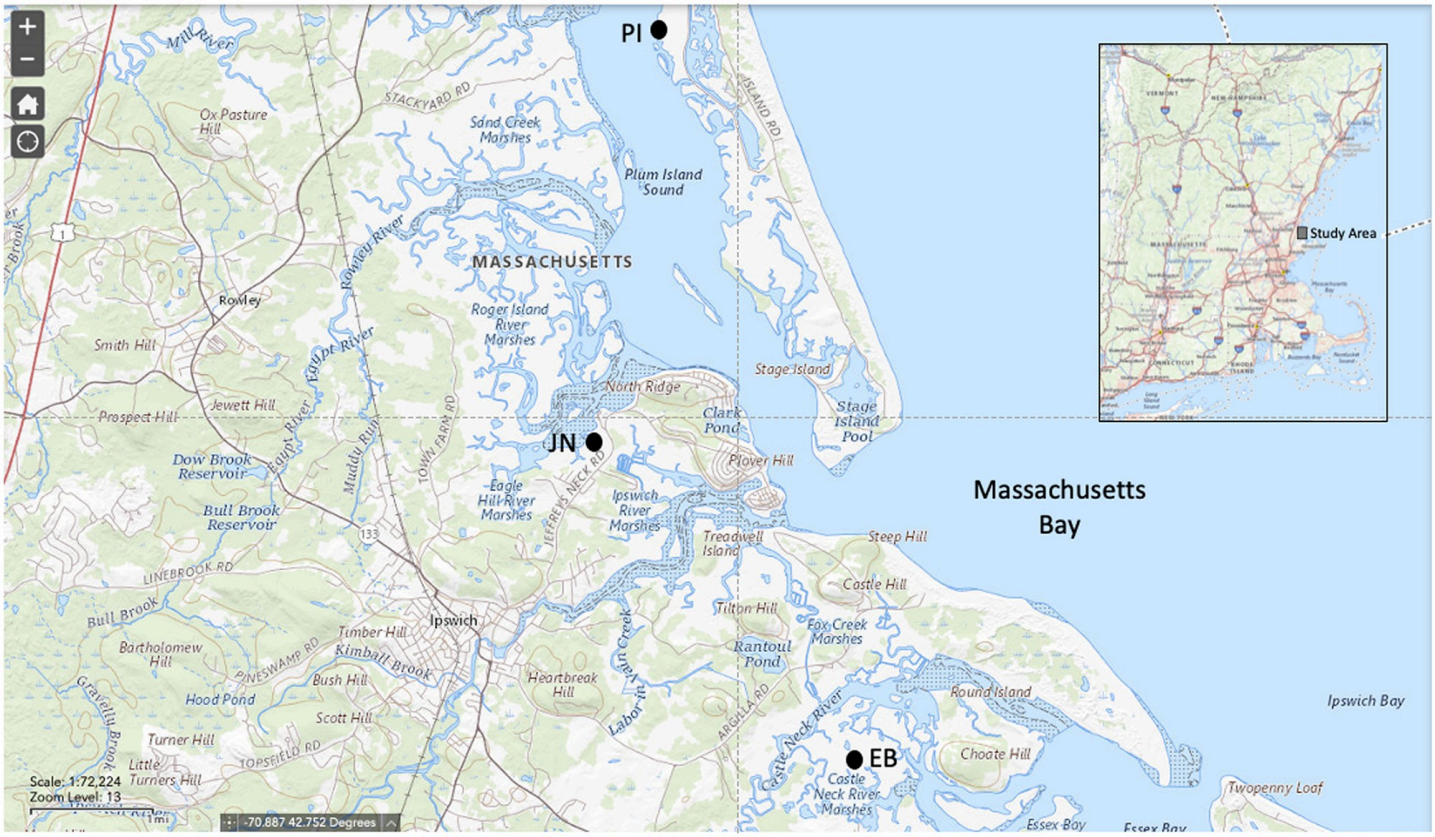
Study sites in the Great Marsh, Massachusetts: Essex Bay (EB), Jeffrey’s Neck (JN), and Plum Island (PI).

**Fig 2:**
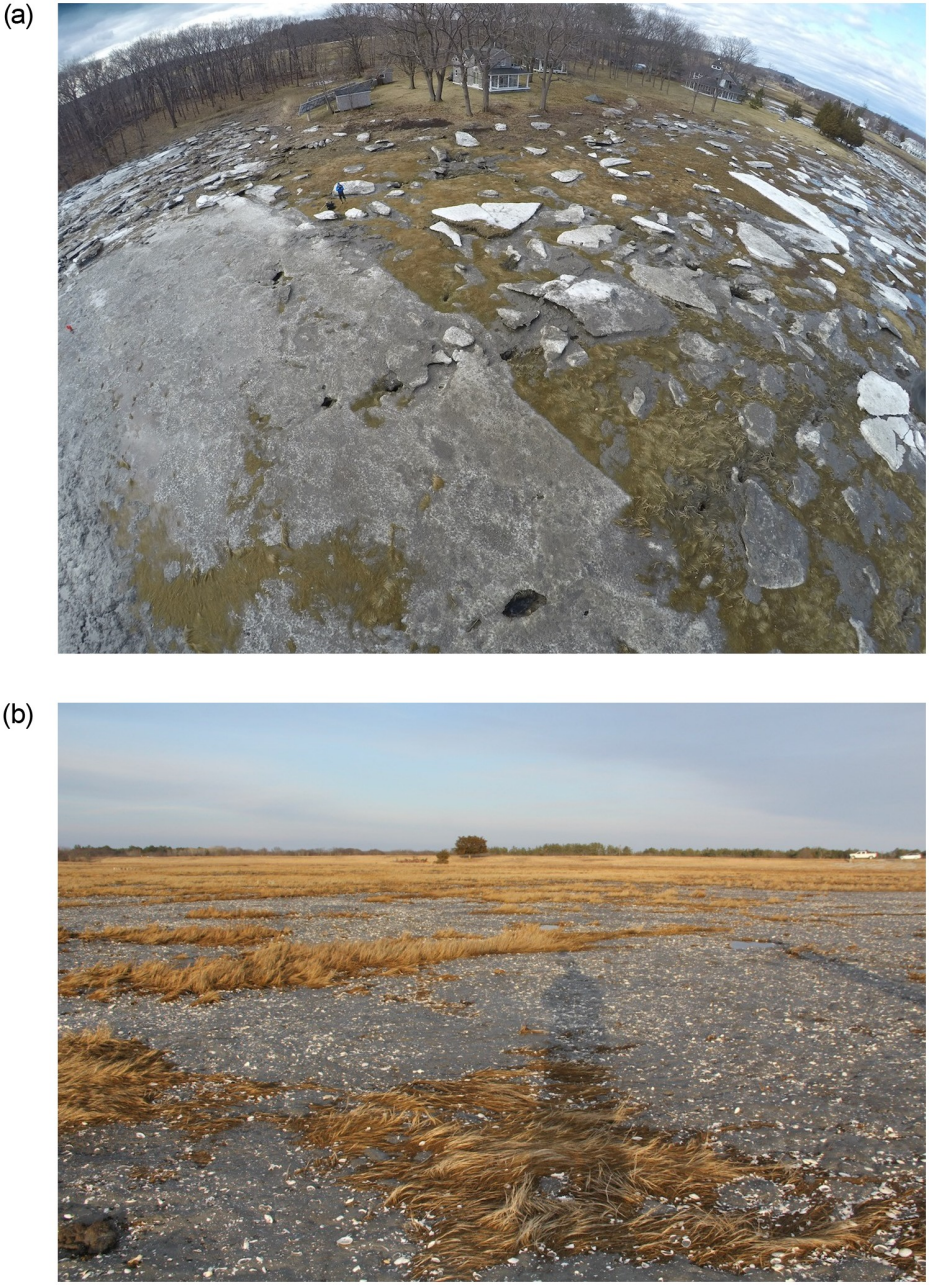
Sediment deposition on the Great Marsh, MA following winter storm Greyson. (a) Extensive fields of layered ice and sediment on the marsh platform. (b) Bivalve shells in the deposited layer of sediment.

In mid-winter 2018, natural deposition of unprecedented scale was observed on salt marshes on the North Shore of Massachusetts and on a smaller scale in New Hampshire and southern Maine. Three areas of contiguous deposits were studied in the Great Marsh (Fig 1). Deposited sediment was observed following winter storm Greyson, which featured a period of abnormally low temperatures combined with high tides and strong winds. Extensive acres of imbricated chunks of layered ice and sediment were documented in the field days after the storm (Fig 2a). These observations, combined with documentation of bivalve shells littering sediment across the high marsh platform (Fig 2b), confirm that ice rafting was the mechanism of sediment delivery. While overwash events from severe storms have been known to deposit thick sediment layers on back-barrier marshes [17], and ice rafting can float slabs of marsh peat onto the high marsh platform from time to time, sediment deposition of this magnitude caused solely by ice rafting has not been documented in Massachusetts. Moreover, it is unknown how large-scale deposition affects marsh plant communities in New England.

Accordingly, the goals of this study were to: 1) document the scale and distribution of this natural sediment event in a salt marsh on the north shore of Massachusetts; and 2) examine the effect of sediment deposition on marsh plant cover and pore water chemistry over the range of sediment thicknesses across three marsh sites. To meet these goals, a combination of aerial drone surveys and field surveys were used, as well as plant assessments on the marsh surface and chemistry analysis of pore water collected from the marsh peat.

## Materials and methodss

### Study sites

The study sites occur in three sections of the Great Marsh on the north shore of Massachusetts (Fig 1). The Great Marsh is the largest contiguous area of salt marsh in New England, with tidal marshes covering an area of about 40 km^2^. The three study sites are spaced across the Great Marsh Estuary in Essex Bay (EB) in the town of Essex, Jeffrey’s Neck (JN) in Ipswich, and Plum Island (PI) in Newbury. These sites were selected because each represented concentrated areas of fairly continuous, and thus well-defined sediment deposition areas facilitating comparison to adjacent portions of salt marsh that did not receive natural sediment additions. Each site contained both high and low marsh habitats within the study area.

### Aerial imagery and spatial analysis

Aerial imagery was used to determine the distribution and area of deposited sediment at each site. Imagery was captured in mid-winter and early spring of 2018. EB and JN were first to be flown via unmanned aerial vehicle (UAV) on February 21^st^, while PI was flown on March 1^st^ due to a brief delay in obtaining necessary permissions for UAV use in the Parker River National Wildlife Refuge. Visible light (RGB true color) geo-tagged imagery of the sediment event areas was acquired at an altitude of 60 m at an effective 1.67 cm ground resolution using *MAPIR Survey3* (MAPIR Inc, San Diego, CA) camera flying on a *3DR Solo* UAV platform (3D Robotics, Berkeley, CA). Flight planning and control for UAV flights used *3DR Tower* software. Programed survey flight lines insured 70–80% overlap with neighboring images to increase resolution and facilitate subsequent photomosaic production using *Pix4DMapper* software version 3.0.18 (Pix4D LLC, Lausanne, Switzerland).

Once the RGB imagery was combined to form a single data layer, a supervised classification was completed to identify and quantify sediment coverage using *ArcGIS* software (ESRI, Redlands, CA) interactive classification methods. This classification used forty-two ground-truthed training sites within each site’s photomosaic image to capture unique signatures in the sediment deposition areas, including determination of the dominant plant community, sediment presence/absence, and other features within each training site. The location of each training site was marked with a Leica GSSN Rover model GS14 Real Time Kinematic (RTK) GPS (Leica Geosystems, St. Gallen, Switzerland) with an accuracy of ±1.5 cm. Additional refinements to the classification were completed with ARIS Grid & Raster Editor, a third-party extension for ArcGIS, ArcMap software (ESRI, Redlands, CA).

### Plot establishment and sediment measurements

A series of randomly located, linear transects were established at each site with a minimum of three and maximum of six depending upon the shape and size of the deposition event at each site. As with transects, the total number of plots varied by site such that EB = 28, JN = 45, and PI = 32). Plots were situated along each transect exactly 20 m apart to avoid sampling bias. At each plot, the thickness of deposited sediment was measured to the nearest mm in the winter and summer of 2018. Plots containing no deposited sediment served as controls. To measure bulk density, cores containing only the surface deposition material were collected at each sedimented plot during the winter sampling event using a sediment corer and stored at 4°C until gravimetric analysis.

### Pore water analyses

Soil pore water was sampled at a depth of ~25 cm at each station using the sipper method [22] which extracts water trapped in pore spaces using a 1mm diameter stainless steel tube fitted with a 60 cc plastic syringe. The depth of ~25 cm is consistently within the original marsh surface, accessing pore water associated with live roots of the existing plant community. Pore water salinity was determined in the field using a Thermo Scientific Orion Star A329 Portable Multiparameter Meter with DuraProbe conductivity cell, while redox potential and pH were obtained using a platinum electrode and a Ross Sure-Flow temperature corrected pH triode, respectively (Thermo Fisher Scientific, Waltham, MA). Because pH varied greatly between plots (3.0–7.3), redox potential was adjusted to pH 5 using the equation Eh_5_ = redox+[(pH − 5)*59]+244 [23].

### Vegetation metrics

Vegetation sampling was completed in late July through early August to represent peak plant growth. Percent cover was recorded using visual estimation assuming a single canopy layer (maximum of 100%) within 0.5 m^2^ plots at each station (total n = 105). Vascular plants were identified to the species level in the field. Bare/unvegetated ground was accounted for in the percent cover estimate and the predominant habitat type was noted in the field (i.e., high marsh or low marsh (below mean high water); [24]).

### Data analysis

Google Earth historical imagery from 2017 was used to identify plots that fell upon areas that were completely or largely unvegetated before the sediment event (e.g., pools and ditches, n = 10) or were located in the upland (n = 2). Because our focus was on impacts to existing vegetation, these plots were omitted from plant and associated diagnostic soil chemistry analyses. However, all plots were included in soil thickness and bulk density measurements except for the two upland plots. To determine whether sediment thickness differed significantly between winter and summer, a two-tailed paired sample t-test was used. Sediment deposition was separated into four thickness categories (0 mm, 1–19 mm, 20–39 mm, and 40–90 mm). One-way ANOVA was used to analyze the effect of thickness category on the change in sediment thickness between winter and summer (S1 Table). Two-way ANOVA tests were used to analyze effects of thickness category on pH (blocked by site), salinity (blocked by habitat type), and percent vegetation cover (blocked by habitat type). Initially, the blocking factors of site, habitat type, and interactive effects were included in each ANOVA model but were removed if p > 0.05. Tukey’s HSD post-hoc test was used to determine differences among treatments. The Shapiro-Wilk goodness-of-fit test was used to determine whether residuals met the assumption of normality. The following transformations were made to satisfy assumptions of parametric tests: log(salinity), log(-pH + 8), and log(-percent cover + 101). Residuals for the ANOVA on percent cover did not pass the Shapiro-Wilk test for normality (p = 0.03) even after data were transformed. However, ANOVA was still used because the distribution of residuals appeared approximately normal, and ANOVA is robust to minor violations of the normality assumption [25]. The Shapiro-Wilk test is also overly sensitive at high sample sizes and should be used in conjunction with graphical approaches for assessing normality. As an added precaution to avoid type I error, the criterion for significance in the ANOVA model was reduced to α < 0.01. When transformations were ineffective in producing approximately normal distributions and homoscedasticity, Wilcoxon rank sum was used for t-tests (controls vs. sediment for salinity) and Kruskal Wallis was used instead of one-way ANOVA (effect of site on Eh). All statistical analyses were performed in JMP Pro 14 (SAS Institute Inc., Cary, NC).

## Results

### Sediment estimates

**Fig 3:**
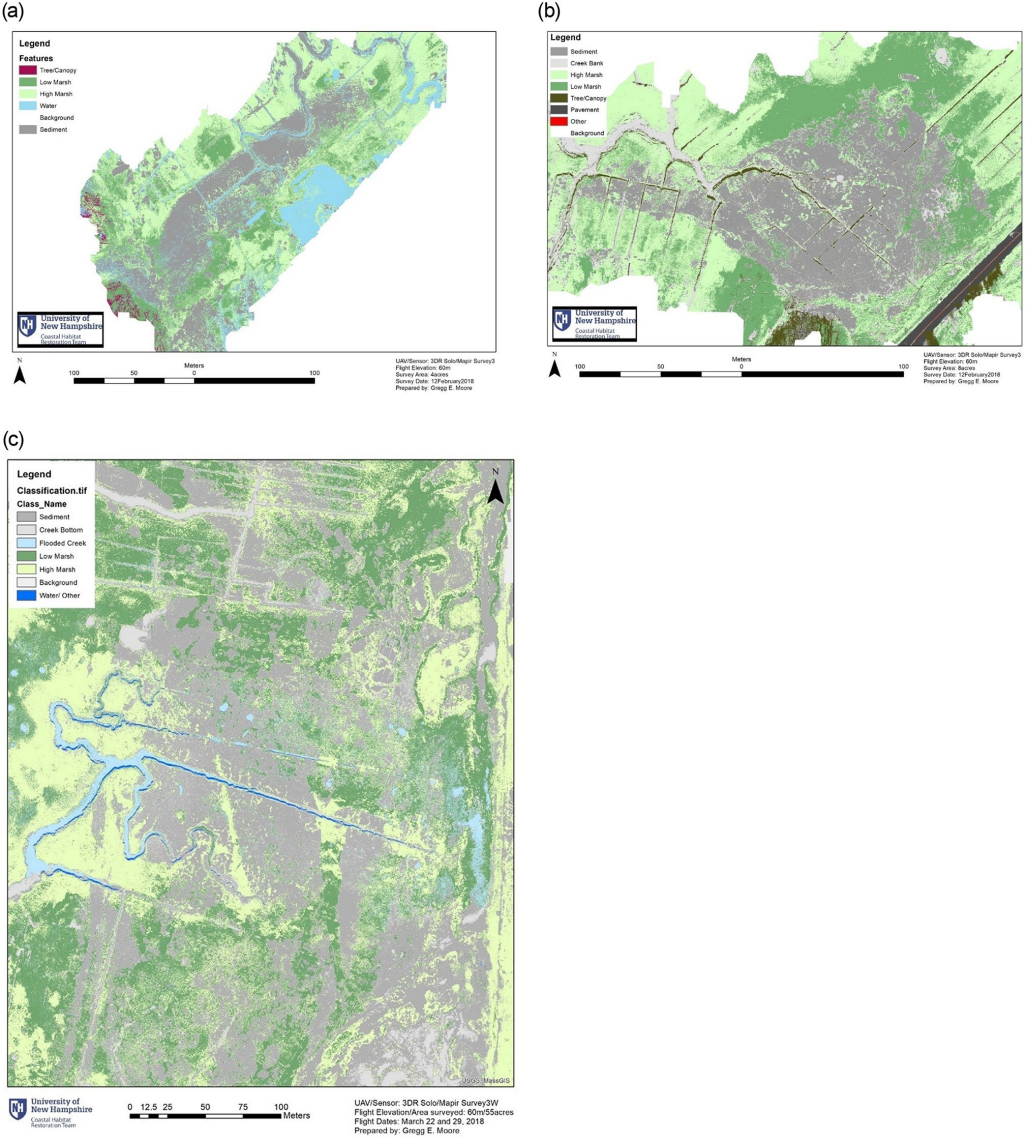
Sediment area supervised classification map. (a) Essex Bay (b) Jefferies Neck (c) Plum Island.

A total of 29.2 ha of naturally deposited sediment was calculated from the orthomosaics and resulting supervised classifications (Fig 3). This total is comprised of 1.7 ha at EB, 3.2 ha at JN, and 24.3 ha at PI (Table 1). The estimates do not account for the entire event, but they capture the most densely covered areas and thus the majority of deposition at each site.

**Table 1:** Comparison of area and sediment characteristics by site.

Site	Area (ha)	Thickness (mm)		Volume (m^3^)	Bulk Density (g/cm^3^)	Total Sediment Mass (kg)
		Winter	Summer			
Essex Bay (EB)	1.7	19.1±2.2	20.9±2.4	355	0.97±0.09	0.34 x 10^6^
Jeffrey’s Neck (JN)	3.2	38.5±3.3	39.6±2.8	1,270	0.82±0.06	1.04 x 10^6^
Plum Island (PI)	24.3	25.6±2.9	26.5±3.8	6,440	1.08±0.09	6.96 x 10^6^
**Total** or ***Mean***	**29.2**	***30*.*1±2*.*1***	***31*.*3±2***	**8,060**	***0*.*92****±****0*.*04***	**8.34 x 10**^**6**^

**Fig 4:**
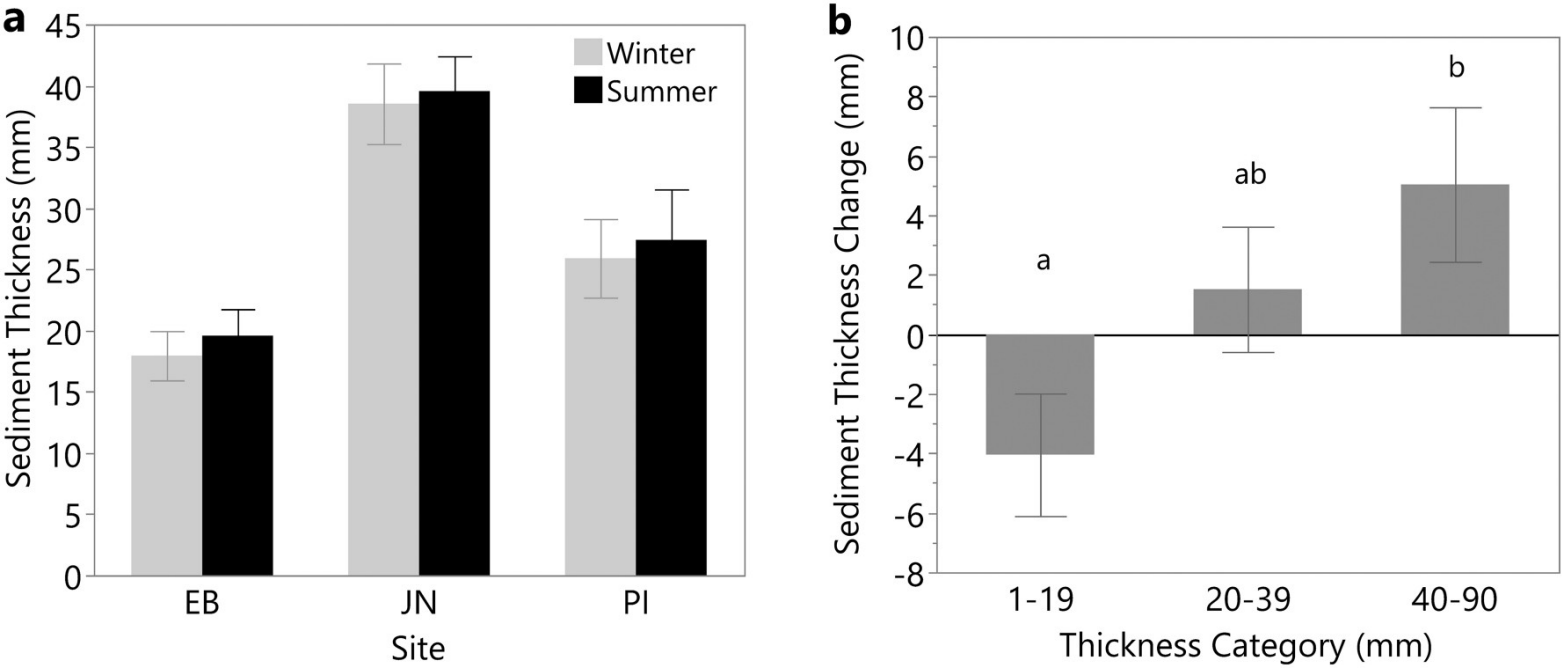
(a) Sediment thickness at each site in winter and summer. (b) The change in thickness of deposited sediment between winter and summer of 2018 for each thickness category. Different letters denote significant differences among thickness categories (Tukey’s HSD). Error bars show standard error.

Winter measures of sediment thickness (30.1±2.1 mm) did not differ significantly from summer measures (31.3±2 mm). However, thickness appeared to differ between sites in both winter and summer (Fig 4a), with sediment at JN being almost double the thickness of EB in both cases. When data were sorted by thickness range classes, there was a significant difference in sediment thickness change (F_2,77_ = 3.6, p = 0.03); for the low thickness plots, the sediment dissipated between winter and summer whereas for higher thickness levels the sediment expanded (Fig 4b). Based on an average summer thickness of 31.3±2.0 mm, the affected areas received a total volume of 8,060 m^3^ of sediment (8.15 x 10^6^ kg; Table 1).

### Pore water chemistry

Sediment deposition had no significant effect on pore water chemistry (Table 2). Deposition, regardless of thickness was not found to elevate salinity. Even if only high marsh data are examined, control salinity does not differ from sedimented areas. However, high marsh areas had significantly higher salinity than low marsh areas (F_1,88_ = 10.3, p = 0.02). Similar to salinity, pH did not differ between treatment and control, but did vary considerably by site with JN showing significantly higher pH than EB and PI (F_2,90_ = 19.8, p<0.001). pH was especially low at PI, with an average of 5.8±.2. Redox potential did not differ between treatment and control but did vary considerably by site (Kruskal Wallace, p<0.001). PI had higher but variable Eh_5_ (139±17 mV) than EB and JN which were consistently anaerobic (12±4 mv and 41±6mV, respectively).

**Table 2:** Pore water characteristics at each site. Means are shown with standard error in parenthesis.

Sediment Thickness (mm)	n	Salinity (psu)	Eh_5_ (mV)	pH
*EB*				
Control	8	32.8 (0.5)	14 (5.6)	6.2 (0.1)
1–19	9	33.8 (1.0)	9 (9.8)	6.3 (0.1)
20–39	9	33.2 (1.6)	13 (4.4)	6.4 (0.1)
40–90	2	31.7 (2.3)	19 (20.9)	6.3 (0.1)
*JN*				
Control	7	34.5 (1.4)	31 (2.8)	6.8 (0.1)
1–19	3	36.1 (2.5)	28 (11.1)	6.5 (0.2)
20–39	13	36.3 (1.3)	39 (6.7)	6.8 (0.1)
40–90	16	34.4 (0.5)	48 (12.3)	6.7 (0.1)
*PI*				
Control	8	34.4 (0.5)	138 (16.9)	6.2 (0.2)
1–19	8	32.0 (1.1)	149 (41.4)	5.6 (0.4)
20–39	5	33.4 (1.3)	140 (37.4)	5.6 (0.5)
40–90	5	33.6 (1.2)	123 (44.4)	5.9 (0.7)

### Vegetation response

**Fig 5:**
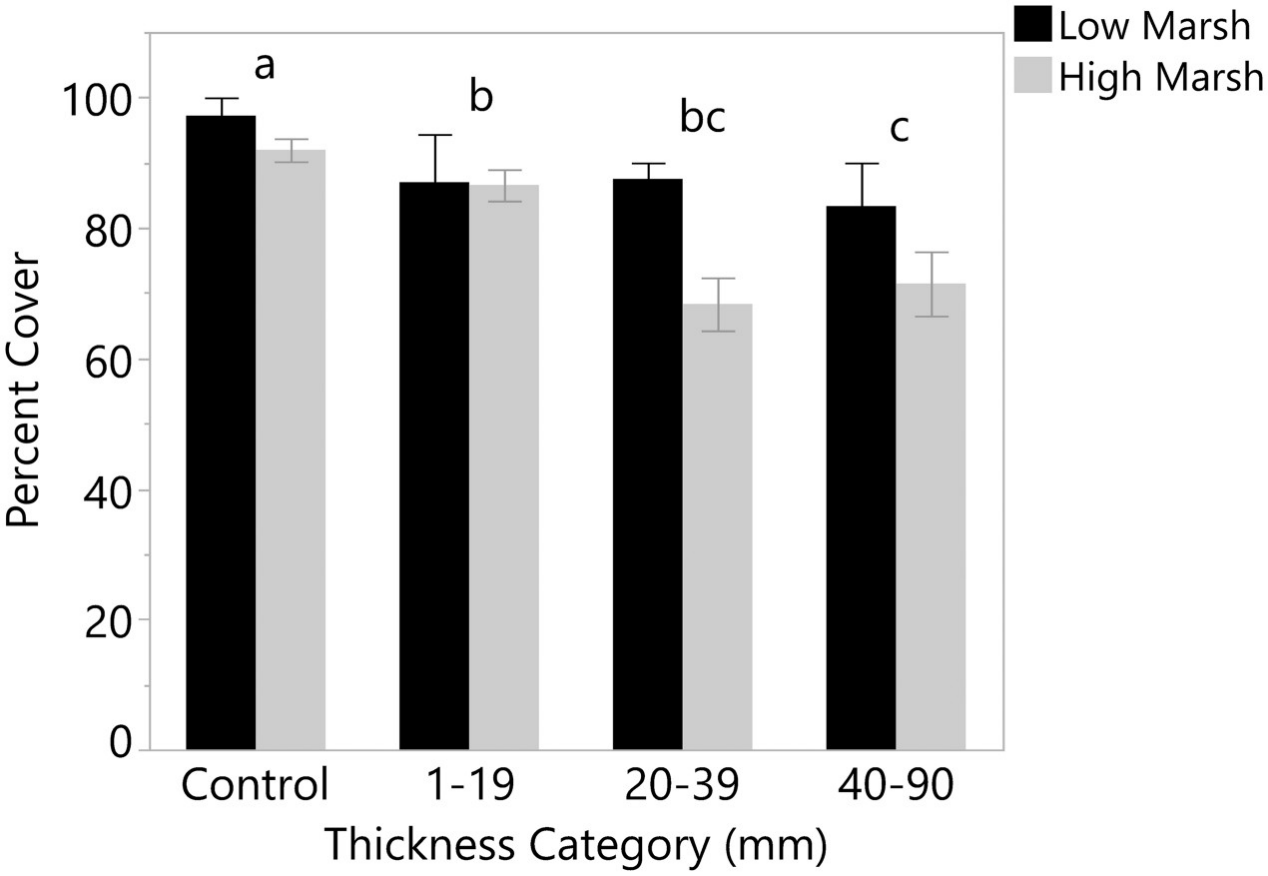
Cover (%) of marsh vegetation for sediment thickness categories separated by habitat type. Different letters denote significant differences among thickness categories (Tukey’s HSD). Error bars show standard error.

The native marsh community appeared to be influenced by sediment additions. Percent cover was reduced in areas receiving sediment after one growing season; percent cover in control plots averaged 93.1±1.6 while cover in sediment plots was 75.6±2.3%. There was a significant effect of sediment thickness (F_3,88_ = 22.1, p < 0.001), with the thickest sediment impairing vegetation more than the thinnest sediment (Tukey’s HSD, Fig 5). All thickness levels had significantly lower cover than controls. High marsh areas had lower cover than low marsh areas (F_1,88_ = 7.8, p = 0.01), and this difference appeared to increase as sediment thickness increased, though the habitat type by sediment thickness interaction was not significant (and was removed from the model).

## Discussion

Storm overwash events are known to contribute significantly to marsh accretion [16, 26]. Although rare in New England, episodic sediment events have been recorded such as the 1938 Hurricane, evidence of which is preserved within the peat profile of many area marshes [27]. The suggestion that natural depositional events can benefit marshes underscores the premise for anthropogenic sediment placement strategies (TLP) to build marsh resilience to sea-level rise. However, the potential ecological benefits of sediment placement must be balanced against the potential for unintended consequences. The goal of management studies examining impacts from TLP therefore must focus on determining the presence and extent of ecological impact–or alternatively to demonstrate evidence that such impacts are minor or insignificant. The present study examines parameters that are known to represent or influence salt marsh health, including vegetation and pore water chemistry to determine effects of deposition resulting from a large-scale ice rafting event.

Other studies have shown that ice rafting plays an important role in marsh accretion in New England [18, 20] and Canada [19], but marsh-wide deposition of this scale associated with ice rafting has not been documented in New England. Dionne [19] reported a total volume of ice-rafted peat clumps of up to 99 m^3^ in a 2500 m^2^ area in Quebec, whereas Argow et al. [18] found ice rafted sediment from adjacent bay and creek bottoms in Essex MA averaged between 132 kg (mode) and 636 kg (mean) sediment per hectare. These authors then calculated the peat thickness that would be expected to develop from this amount of sediment mixed with plant organic matter to estimate a 2 to 11% annual contribution from winter ice events for the entire marsh. Working along the Maine coast, Wood et al. [20] showed that ice-rafted material can contribute from 0–100% of the accretion in marshes. These studies measured discrete patches of deposition whereas our study was able to measure widespread, fairly even deposition at three marshes averaging 279,000 kg/ha.

Overall, the deposited sediment documented in this study did not significantly change in area or thickness during the 6–7 month study period. However, when separated by thickness category, the thinnest sediment (1–19 mm) decreased somewhat over time, likely due to erosion into channels or washing through previous years vegetation to the soil surface. In contrast, the thickest sediment became thicker, possibly due to spring plant growth pushing up the layer of sediment or due to shoot and root ingrowth (as seen in [28]). Newly deposited sediment may compact as it consolidates [16, 28, 29], but this was not found in our study. Since the mechanism of sediment addition in our study was ice rafting instead of storms or TLP, the weight of the overlying ice could have already compressed the deposited sediment before thickness was measured.

Porewater measurements showed that soil conditions were similar between sedimented areas and controls. In Gulf Coast studies, thick sediment addition resulted in higher Eh compared with controls when applied at thicknesses greater than 100 mm [30] or 150 mm [31]. Mendelssohn and Kuhn [31] found that salinity was also higher in areas receiving >150 mm of sediment than areas receiving no sediment. Our results in temperate marshes with larger tidal ranges show that sediment deposition of <100 mm had no effect on soil Eh or salinity, suggesting that sedimented areas and controls were equally hospitable for marsh plants.

Large-scale sediment deposition may have negative impacts on plant communities in the short term, but the impacts were relatively small in our study, with plant cover in sedimented areas reaching 75% of controls within one growing season. Since pore water chemistry was similar between sedimented areas and controls, there is no evidence that soil conditions caused the lower cover found in sedimented areas. Instead, plant cover may have been lower in sedimented areas simply due to the energetic cost to plants from having to penetrate the added layer of sediment. Since the deposition event occurred during winter, burial of winter shoots could have also prevented diffusion of oxygen into the roots, resulting in further plant stress or mortality [32]. Grain size or organic matter content may affect soil characteristics and influence plant recovery following deposition [31].

While plant cover was reduced by sediment deposition after one growing season in this study, benefits to plants are well-documented in southern marshes. Studies on *S*. *alterniflora* marshes in the southern U.S. have shown that TLP resulted in greater aboveground growth compared to submerging areas that did not receive sediment [21, 31, 33–37]. Marshes that were not submerging have also been shown to benefit from sediment addition through improved aboveground growth [15, 37, 38]. Benefits to plant growth from TLP are attributed to higher redox potential and lower sulfide levels, which both indicate a reduction in flooding stress [31]. Sediment addition can also temporarily boost growth through the sudden infusion of nutrients contained in the sediment [31, 36]. Although sediment addition may reduce aboveground growth in the short-term, marsh plants in the Gulf have been shown to fully recover within one year [16, 21].

The effect of sediment addition on plants may vary depending on the sediment thickness [30, 34, 36]. While percent cover in all sediment treatments was lower than in controls in this study, the lowest thickness (1–19 mm) had greater plant cover than the other thickness levels. Similarly, Walters and Kirwan [38] found that their lowest thickness treatment (50 mm) had the greatest benefit to *S*. *alterniflora* growth, with thicker sediment resulting in a diminishing effect or even plant mortality when thickness exceeded 300 mm in a mesocosm study in Virginia. Also showing the importance of sediment thickness, Reimold et al. [39] found that burial exceeding 210 mm killed *S*. *alterniflora* in Georgia, leaving the slower process of colonization through seeds as the only means for recovery. Marsh elevation also likely influences plant responses to burial, as suggested by the higher cover in low marsh sediment plots than high marsh sediment plots in this study.

The sediment deposition documented in this study could improve marsh resilience to sea level rise in the long term. Surface Elevation Tables (SETs) indicate that nearby marshes in New Hampshire are losing elevation relative to sea level rise at a rate of ~2 mm/year [12]. In the Great Marsh, the average rate of sediment accretion on the high marsh was determined to be 2.7 mm/year [40]. Based on this rate, the average thickness of the sediment deposition event measured in the Great Marsh (30.1±2.1 mm) amounts to about eleven years of normal annual accretion of sediment. This high-deposition anomaly underscores the importance of collecting long-term accretion data since short-term datasets may fail to capture such an important deposition event. If compaction and subsidence are minimal, the added sediment should reduce flooding stress to plants by increasing marsh elevation.

The results of this study provide insight as to how marsh plants in Massachusetts could respond to a modest application of TLP. Our data suggest that plants would recover to 75% of the cover found in untreated areas within one growing season if sediment was applied to the marsh at an average thickness of 30 mm. However, longer time periods are needed for full recovery, and it is unknown whether TLP can prevent conversion of high marsh to low marsh or overall marsh loss.

## Conclusions

While it is unclear how climate change will impact the frequency of ice rafting events in the future, sediment delivery by ice likely plays a large and perhaps underappreciated role in building marsh elevation in New England. Although rare in Massachusetts, large-scale sediment deposition through ice rafting results in a sudden influx of elevation capital and accounted for up to 11 years of typical sediment accretion over just one winter. Our results show that deposited sediment reduced plant cover by 17% in the short term, but plants are expected to fully recover within 1–2 growing seasons based on previous studies. Similar impacts could be expected from TLP, but the method of sediment delivery (e.g., jet spray of dredged material) may affect plant responses differently. An alternative approach could add sediments with light track machinery when the marsh is frozen and covered with ice. Further monitoring and research are needed to determine how large-scale sediment deposition, both natural and artificial, will affect long-term marsh resilience to sea level rise in New England.

## Supporting information

S1 Table Statistics tables for analyses on sediment thickness, soil parameters, and percent cover of halophytes. Initially, the blocking factors of site, habitat type, and interactive effects were included in each ANOVA model but were removed if they had a p > 0.05; no interactive effects were significant.
(DOCX)

S2 Table Raw data on sediment deposit thickness in winter and summer 2018, bulk density, salinity, redox, pH and percent cover of salt marsh plants. EB = Essex Bay, JN = Jeffrey’s Neck, and PI = Plum Island.
(XLSX)
