# Supplementary material for: Effects of a large-scale, natural sediment deposition event on plant cover in a Massachusetts salt marsh
Source: PLoS One. 2021 Jan 22;16(1):e0245564. doi: 10.1371/journal.pone.0245564 (PMC7822311; doi:10.1371/journal.pone.0245564)
Supplement: S1 Table — Initially, the blocking factors of site, habitat type, and interactive effects were included in each ANOVA model but were removed if they had a p > 0.05; no interactive effects were significant. (DOCX) [file pone.0245564.s001.docx]

|  | **df** | **SS** | **F Ratio** | **p value** |
| --- | --- | --- | --- | --- |
| **Sediment Thickness** (Paired t-test) |  |  |  |  |
| Winter vs. Summer | 79 |  | 0.859^A^ | .393 |
|  |  |  |  |  |
| **Sediment Thickness Change** (ANOVA) |  |  |  |  |
| Thickness Category | 2 | 1005.9 | 3.578 | .0327 |
|  |  |  |  |  |
| **Salinity**^B^ (ANOVA) |  |  |  |  |
| Habitat (High vs. Low) | 1 | 0.0893 | 10.378 | .002 |
| Thickness Category | 3 | 0.0139 | 0.538 | .658 |
|  |  |  |  |  |
| **pH**^C^ (t-test) |  |  |  |  |
| Treatment (sediment vs control) | 39.8 |  | -0.171 | .865 |
| **pH**^C^ (ANOVA) |  |  |  |  |
| Site | 2 | 2.9406 | 15.300 | <.0001 |
| Thickness Category | 3 | 0.1408 | 0.4885 | 0.691 |
|  |  |  |  |  |
| **Eh** (Wilcoxon Rank Sum) |  |  |  |  |
| Treatment (sediment vs control) | 1 |  |  | .507 |
|  |  |  |  |  |
| **Eh** (Kruskal Wallace Test) |  |  |  |  |
| Site | 2 |  |  | <.0001 |
| **Percent Cover**^D^ (ANOVA) |  |  |  |  |
| Habitat Type (High vs Low) | 1 | 5.071 | 7.84 | .006 |
| Thickness Category | 3 | 42.925 | 22.13 | <.0001 |
|  |  |  |  |  |

**S1 Table. Statistics tables for analyses on sediment thickness, soil parameters, and percent cover of halophytes***.* Initially, the blocking factors of site, habitat type, and interactive effects were included in each ANOVA model but were removed if they had a p > 0.05; no interactive effects were significant.

^A^ t Ratio

^B^ Log transformed

^C^Transfomed using log(-pH+8)

^D^Transformed using log(-percent cover +101)
